# YTHDF1 shapes immune-mediated hepatitis via regulating inflammatory cell recruitment and response

**DOI:** 10.1016/j.gendis.2024.101327

**Published:** 2024-05-16

**Authors:** Hao Li, Kailun Yu, Xiandan Zhang, Jiawen Li, Huilong Hu, Xusheng Deng, Siyu Zeng, Xiaoning Dong, Junru Zhao, Yongyou Zhang

**Affiliations:** a State Key Laboratory of Cellular Stress Biology, Innovation Center for Cell Signaling Network, School of Life Sciences, Xiamen University, Xiamen, Fujian 361102, China; b National Institute for Data Science in Health and Medicine Engineering, Faculty of Medicine and Life Sciences, Xiamen University, Xiamen, Fujian 361102, China; c School of Pharmaceutical Sciences, Xiamen University, Xiamen, Fujian 361102, China

**Keywords:** Concanavalin A, Hepatitis, Inflammatory response, N^6^-methyladenine, YTHDF1

## Abstract

Severe immune responses regulate the various clinical hepatic injuries, including autoimmune hepatitis and acute viral hepatitis. N6-methyladenosine (m^6^A) modification is a crucial regulator of immunity and inflammation. However, the precise role of YTHDF1 in T cell-mediated hepatitis remains incompletely characterized. To address this, we utilized Concanavalin A (ConA)-induced mouse liver damage as an experimental model for T cell-mediated hepatitis. Our findings found that hepatic YTHDF1 protein rapidly decreased during ConA-induced hepatitis, and YTHDF1-deficient (*Ythdf1*^*−/−*^) mice showed more susceptibility to ConA-induced liver injury, along with an intensified inflammatory storm accompanied by aggravated hepatic inflammatory response via ERK and NF-κB pathways. Interestingly, hepatic-specific over-expression or deletion of YTHDF1 exhibited redundancy in ConA-induced liver injury. Validation in bone marrow chimeric mice confirmed the necessity of YTHDF1 in hematopoietic cells for controlling the response to ConA-induced hepatitis. Additionally, our data revealed that YTHDF1 deletion in macrophages exacerbated the inflammatory response induced by lipopolysaccharide. In summary, our study uncovered that YTHDF1 deficiency exacerbates the immune response in ConA-induced hepatitis by modulating the expression of inflammatory mediators, highlighting the potential of YTHDF1 as a therapeutic target for clinical hepatitis.

## Introduction

Autoimmune hepatitis (AIH) is a severe autoimmune disease with an unclear etiology, characterized by immune-mediated hepatocyte destruction leading to hepatic necroinflammation, cirrhosis, hepatocellular carcinoma, and potential fatality.^1^ T cells play a central role in initiating and perpetuating hepatic damage through self-protein targeting.^2^ The pathophysiology of AIH involves proinflammatory cytokine release from T cells, macrophages, and other inflammatory cells, including IFN-γ, TNF-α, IL-6, IL-12, and IL-23.^3^ Despite the proposed risk factors and therapeutic options, the mechanisms underlying AIH remain incompletely understood, emphasizing the need to unravel its molecular basis for diagnostic and therapeutic advancements. The lectin concanavalin A (ConA) mouse model simulates human immune-induced hepatitis, characterized by elevated serum inflammatory mediators, aminotransferases, and immune cell infiltration.^4^ ConA activates T cells by binding to sinusoidal endothelial cells and Kupffer cells, triggering a cytokine storm, recruiting inflammatory cells within the liver, and causing hepatic injury.^5^^,^^6^ Thus, ConA-induced hepatitis provides a well-established mouse model to investigate the cellular and molecular mechanisms of immune-mediated hepatitis.

N^6^-methyladenosine (m^6^A), a prevalent post-transcriptional mRNA modification, dynamically regulates various cellular processes. Writers (METTL3, METTL14, WTAP), erasers (FTO, ALKBK5), and readers (YTHDF1, YTHDF2, YTHDF3, YTHDC1, YTHDC2) modulate m^6^A, influencing RNA transcription, maturation, localization, function, and metabolism.^7^, ^8^, ^9^, ^10^ Recent research links m^6^A to immunity and inflammation, impacting the development, differentiation, activation, migration, and polarization of innate and adaptive immune cells.^11^, ^12^, ^13^, ^14^ This modification modulates immune responses and contributes to the pathogenesis of immune-related diseases, including infections, inflammatory diseases, cancers, and autoimmune disorders.^11^ Crucially, m^6^A impacts CD4^+^ T cells function. ALKBH5 reduces m^6^A levels in CXCL2 and IFNG mRNA, enhancing mRNA stability and translation, thereby promoting CD4^+^ T cell responses in autoimmunity.^15^ METTL14-mediated m^6^A modification increases splicing and expression of miR-149–3p, influencing Th17 differentiation in enterotoxigenic *Bacteroides fragilis*-induced intestinal inflammation and carcinogenesis.^16^ Additionally, METTL3-mediated m^6^A methylation in CD40, CD80, and the TLR signaling adaptor TIRAP supports dendritic cell maturation and enhances T cell activation.^17^ Deletion of METTL14 in myeloid cells exacerbates macrophage responses to acute bacterial infection by targeting SOCS1 in an YTHDF1-dependent m^6^A manner.^18^ However, the links of m^6^A and its regulators to AIH remain poorly understood.

In this study, we addressed the unresolved aspect of m^6^A and its regulators in T cell-mediated hepatitis, despite their well-established functions in immunity, viral infection, and autoinflammatory disorders.^12^, ^13^, ^14^^,^^19^^,^^20^ Interestingly, our findings revealed a significant correlation between hepatic YTHDF1 protein changes and acute immune-mediated hepatitis for the first time. Depletion of YTHDF1 intensifies liver injury upon ConA challenge, leading to a pronounced cytokine storm and severe hepatic inflammatory response, primarily via the extracellular signal-regulated kinase (ERK) and NF-κB pathways. Notably, YTHDF1 in hepatocytes seems redundant, whereas its presence in hematopoietic cells is crucial for the immune response and T cell-mediated liver injury triggered by ConA. Furthermore, our study demonstrates that YTHDF1 deletion in macrophages exacerbates lipopolysaccharide (LPS)-induced inflammatory responses, emphasizing the necessity of YTHDF1 in immune cells for an effective inflammatory response. Overall, our results underscore the pivotal role of YTHDF1 in ConA-induced hepatitis, positioning it as a promising therapeutic target for immune-mediated hepatitis.

## Methods and materials

### Mice

C57BL/6 YTHDF1 knockout (*Ythdf1*^−/−^) mice, YTHDF3 knockout *(Ythdf3*^−/−)^ mice, and Rosa26-LSL-Cas9-tdTomato (*Cas9*^*+/−*^) mice were obtained from Cyagen Bioscience and Gempharmatech Inc. Wild type (WT), *Ythdf1*^−/−^, *Ythdf3*^−/−^, and *Cas9*^*+/−*^ mice on C57BL/6 background were bred and housed in specific pathogen-free conditions at the Xiamen University Laboratory Animal Center. Mice at 6–10 weeks of age were used for experiments.

### ConA-induced hepatitis

Mice were intravenously injected via the tail vein of Concanavalin A (Sigma, Cat# C2010) (ConA) at sublethal doses of 8 mg/kg or 10 mg/kg body weight, and were subsequently sacrificed at various time points (1, 3, 8, 24 h) following ConA administration. Survival testing was performed with ConA at a lethal dose of 20 mg/kg.

### Adeno-associated virus (AAV) associated hepatic specific gene expression

To generate hepatic-specific *Ythdf1* knockout mice, we combined the technology of CRISPR/Cas9 and Flox-Cre system depending on AAV.^21^^,^^22^ The human thyroxine-binding globulin (TBG) promoter and the AAV2/8 serotype were used to drive hepatocyte-specific gene expression. For knockout of YTHDF1, AAV2/8-TBG-Cre-U6-sg*Ythdf1*, including two sgRNAs targeting YTHDF1, was injected into Rosa26-LSL-Cas9-tdTomato C57BL/6 mice. The *Ythdf1* sgRNA sequences (sg*Ythdf1*#1: 5′-GCTTCATGAACAGCGCGGCG-3′; sg*Ythdf1*#2: 5′-GGTAAGGATCACTCATCGAG-3′) were synthesized, annealed, and subcloned into a modified pAAV-TBG-Cre-U6-sgRNAs vector. To induce the hepatic-specific overexpression of *Ythdf1*, the pAAV-TBG-m*Ythdf1* (Mouse *Ythdf1*, protein id = NP_776122.1) plasmid vector was conducted for gene expression in WT mice. To generate AAV of serotype 2/8, AAV-293 cells (Procell, Cat# CL-0019) were co-transfected with pAAV-TBG-Cre-U6-sg*Ythdf1*#1-U6-sg*Ythdf1*#2 or pAAV-TBG-m*Ythdf1*, pAAV2/8 (addgene, Cat# 112864) and pAdDeltaF6 (addgene, Cat# 112867). At 60 h post-transfection, cells and culture medium were harvested and processed separately. The medium was mixed with PEG8000 at 4 °C overnight and then precipitated by centrifugation. Cells were lysed in the lysis buffer with three of freeze–thaw cycles for virus release. The crude viruses combined cell lysis with medium precipitate were purified using discontinuous iodixanol (Sigma, Cat# D1556) gradient ultracentrifugation and concentrated by Amicon Ultra centrifugal filter (Merck, 100K, Cat# C134281). The AAV titer was determined by qPCR using primers targeting the TBG promoter on the AAV vector, and the AAV purity was tested by silver staining. For animal infection, the 6-week-old male mice were injected with 2 × 10^11^ genome copies of AAV per dose via the tail vein.

### Bone marrow transplantation (BMT)

Eight-week-old WT or *Ythdf1*^*−/−*^ mice were received irradiation with 8 Gy X-rays. Six hours later, bone marrow cells from *Ythdf1*^*−/−*^ or WT mice were transplanted into the irradiated mice via intravenous injection at a number of 200E4 cells per mouse. In brief, mouse bone marrow cells were flushed out from the femur and tibia, followed by the removal of red blood cells. The transplantation efficiency was assessed by genotyping of blood and tail samples, as well as by Western blotting of spleen and liver samples four weeks after transplantation.

### Isolation and culture of primary peritoneal macrophages

Peritoneal macrophages (PM) were acquired using the thioglycollate elicitation as described before.^23^ The isolated PM were cultured in RPMI1640 supplemented with 10% fetal bovine serum (GEMINI, Cat# A24G00J), 100 U/mL penicillin, and 100 mg/mL streptomycin in a humidified 37 °C incubator with 5% CO_2_. For the LPS challenge, macrophages were exposed to 1 μg/mL of LPS (Sigma, Cat# L2630) for various time points, and subsequent cell lysates containing protein or RNA were analyzed.

### Detection of ALT and AST

Blood samples were collected at the indicated time points after ConA injection, and serum levels of alanine aminotransferase (ALT) and aspartate aminotransferase (AST) were determined using ALT Detection Kit (Nanjing Jiancheng, Cat# C009-2-1) and AST Detection Kit (Nanjing Jiancheng, Cat# C010-2-1) according to the manufacturer's instructions.

### Measurement of cytokine levels in the serum

The serum levels of IL-6 (Multisciences, Cat# EK206), TNF-α (Multisciences, Cat# EK282HS), or CXCL-1 (Multisciences, Cat# EK296) were measured using enzyme-linked immunosorbent assay kits purchased from Multisciences according to the manufacturer's instructions.

### Western blotting analysis

The collected hepatic tissues or cell samples were homogenized in RIPA buffer (50 mM Tris (pH 8.0), 15 mM NaCl, 2 mM EDTA, 1% SDS, 0.5% sodium deoxycholate, 1% NP-40) and supplemented with protease inhibitor cocktail (APExBIO, Cat# K1007) and phosphatase inhibitor cocktail (APExBIO, Cat# K1015). Protein concentrations were measured using the BCA protein assay kit (Tiangen, Cat# PA115-02). Approximately 40 μg of protein sample was separated on 8% or 10% gel by SDS-PAGE electrophoresis and transferred to a PVDF membrane (Millipore, Cat# IPVH00010). Membranes were blocked with 5% skim milk in TBST (50 mmol/L Tris, 150 mmol/L NaCl, 0.5 mmol/L tris-buffered saline, and Tween-20, pH 7.5) at room temperature for 1 h. After blocking, the membranes were incubated with primary antibodies at 4 °C overnight, washed with TBST, and then incubated with secondary antibodies at room temperature for 1 h. Finally, the membranes were visualized with ECL Western blotting Substrates (ThermoFisher Scientific, Cat# 34075) via the C-Series Imaging System (Azure Biosystems C500). The signal intensity was quantified using the Image J software. The primary antibodies using Western blotting are as follows: anti-YTHDF1 (Abcam, Cat# ab252346), anti-YTHDF2 (Abcam, Cat# ab246514), anti-YTHDF3 (Abcam, Cat# ab220161), anti-YTHDC1 (ABclonal, Cat# A7318), anti-YTHDC2 (Proteintech, Cat# 27779-1-AP), anti-METTL3 (Abcam, Cat# ab195352), anti-METTL14 (Proteintech, Cat# 26158-1-AP), anti-WTAP (Abcam, Cat# ab195380), anti-FTO (Proteintech, Cat# 27226-1-AP), anti-ALKBH5 (Proteintech, Cat# 16837-1-AP), anti-p-STAT3 (CST, Cat# 9145T), anti-STAT3 (CST, Cat# 9139T), anti-p-STAT1 (CST, Cat# 7649), anti-STAT1 (CST, Cat# 14994), anti-p-ERK1/2 (CST, Cat# 4370T), anti- ERK1/2 (CST, Cat# 4695T), anti-p-IκBα (CST, Cat# 2859), anti-IκBα (CST, Cat# 4814), anti-p-p65 (CST, Cat# 3033), anti-p65 (CST, Cat# 8242), anti-IKKβ (CST, Cat# 8943), anti-p-p38 (CST, Cat# 4511T), anti-p38 (CST, Cat# 8690T), anti-p-JNK (CST, Cat# 4668T), anti-JNK (CST, Cat# 9252T), anti-c-Jun (CST, Cat# 9165), anti-IKK-α/β(CST, Cat# 2697), and anti-β-actin (Sigma, Cat# A5316).

### H&E and immunohistochemistry analysis

For histological analysis, liver specimens were fixed in 4% paraformaldehyde (PFA) overnight and then embedded in paraffin for sectioning (5 μm). The sections were deparaffinized with xylene and rehydrated by a graded series of ethanol concentration (100%, 95%, 85%, 70%, and PBS). Tissue sections were stained with hematoxylin and eosin (H&E) for morphological analysis. For immunohistochemistry analysis, liver sections were boiled with citrate buffer (pH 6.0) for antigen retrieval and then followed the instructions of the UltraSensitive SP kit (MXB biotechnologies, Cat# KIT-9720). Sections were incubated at 4 °C overnight with the primary antibodies, including anti-YTHDF1 (Proteintech, Cat# 17479-1-AP, 1:200), anti-YTHDF2 (Abcam, Cat# ab246514, 1:200), anti-YTHDF3 (Abcam, Cat# ab220161, 1:200), and anti-CD4 (Abcam, Cat# ab183685, 1:1000). After washing with PBST, the tissue sections were incubated with streptavidin anti-mouse/rabbit IgG at room temperature for 10 min and then stained with DAB kit (MXB biotechnologies, Cat# DAB-0031). The Motic VM1 microscope and Motic DSAssistant lite software were used for the panoramic scan of H&E and immunohistochemistry images.

### RNA sequencing (RNA-seq) analysis

Total RNA was isolated from *Ythdf1*^−/−^ and WT liver or PM with RNA simple total RNA KIT (Tiangen, Cat# DP419) according to the manufacturer's instructions. Three independent samples (biological replicates) were established for each group. The RNA sequencing library was prepared and sequenced on a BGISEQ-500 platform (BGI, China) by SE50 sequencing. Sequencing reads were mapped by the STAR aligner to the mouse reference genome, and gene expression counts were calculated with HTSeq. Differential expression analysis was performed using the edgeR package and the pathways enriched in the differentially regulated genes were analyzed by Gene Set Enrichment Analysis (GSEA) (http://software.broadinstitute.org/gsea/index.jsp) according to standard procedures.^24^ All the sequencing data generated in this study are available at NCBI Gene Expression Omnibus (GEO, accession number GSE238229 and GSE238236).

### Statistical analysis

All statistical analyses were performed using GraphPad Prism 8.0. Bioinformatics analysis was performed with R (Version 3.61) (http://www.R-project.org/). The data are presented as the mean ± SD as indicated in the figure legends, and the two-tailed unpaired Student's *t*-test was used to compare the two groups. The survival curves were analyzed using the Log-rank test. The precise value of *n* (number of biological or experimental replicates) is provided in the figure legends. Unless otherwise specified, all values were calculated from at least three independent biological replicates. For all the statistical analyses between the two groups, the results are shown in the figures as follows: ∗*p <* 0.05, ∗∗*p <* 0.01, ∗∗∗*p* *<* 0.001, and ns (not significant).

## Results

### The decreased protein expression of m^6^A reader YTHDF1, YTHDF2 and YTHDF3, during early ConA-induced hepatitis

**Figure 1:**
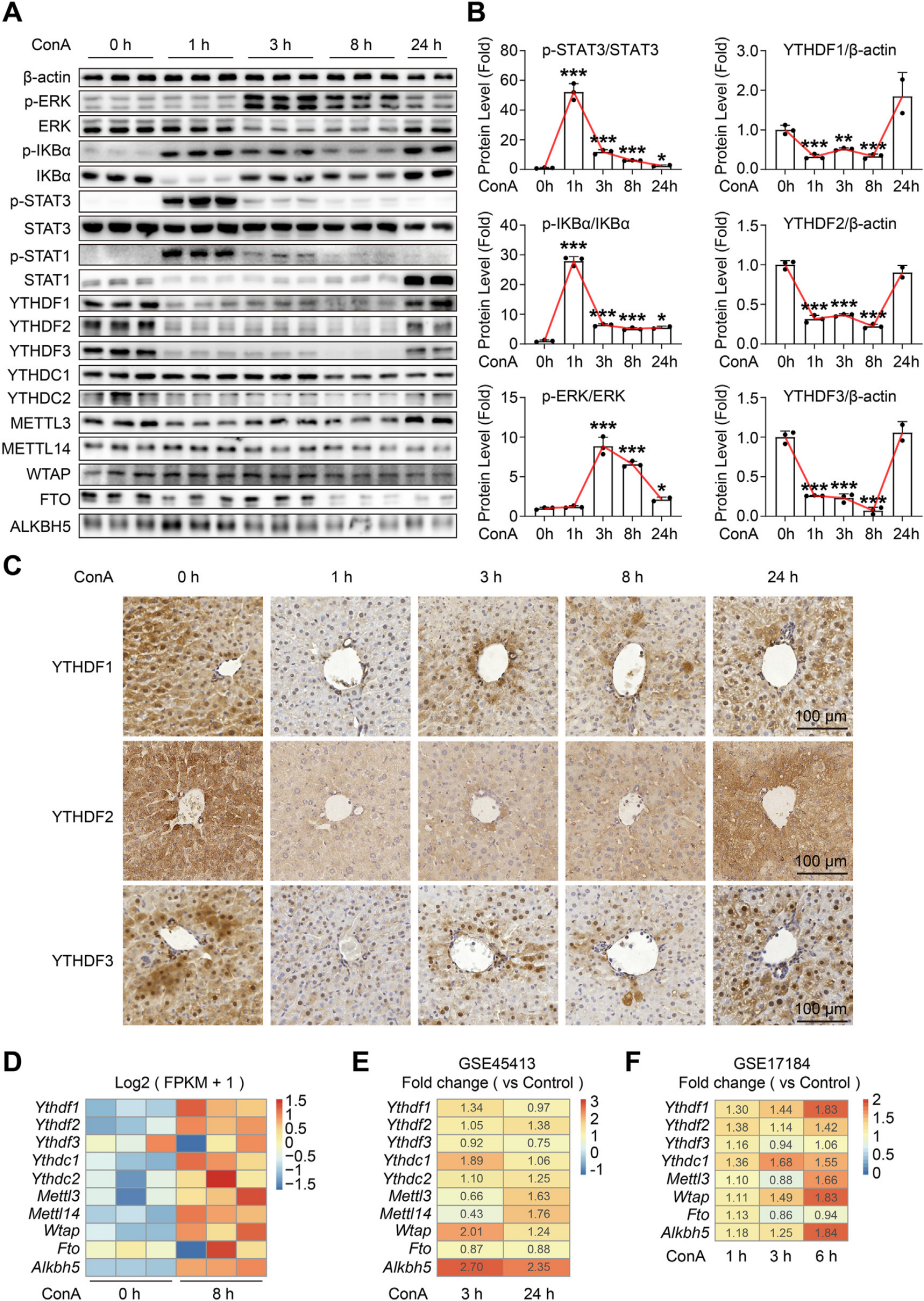
The protein level of m^6^A reader YTHDF1, YTHDF2 and YTHDF3 decrease during the early stage of ConA-induced hepatitis. Eight-week-old male C57BL/6 WT mice were intravenously injected with ConA (10 mg/kg) or saline as vehicle control, and the liver tissues were collected at various time points after ConA treatment, *n* = 2–3 per group. (**A, B**) Immunoblot analysis of liver tissues with m^6^A family and representative inflammatory pathway antibodies (A), followed by gray analysis using Image J (B). **(C)** Representative images of liver sections for immunostaining analysis with YTHDF1, YTHDF2 and YTHDF3 antibodies, Scale bar = 100 μm. **(D)** Heatmap analysis from liver RNA-seq data indicated the mRNA expression level of the m^6^A family after ConA treatment for 8 h. **(E, F)** Heatmap analysis from the GEO database showed the fold change of m^6^A family mRNA levels after the ConA challenge at indicated time points compared with vehicle control in liver tissues. Data are presented as mean ± SD; ∗*p <* 0.05, ∗∗*p <* 0.01, ∗∗∗*p <* 0.001 compared to the control group of WT ConA 0h, based on unpaired two-sided Student's *t*-test.

**Fig. S1:**
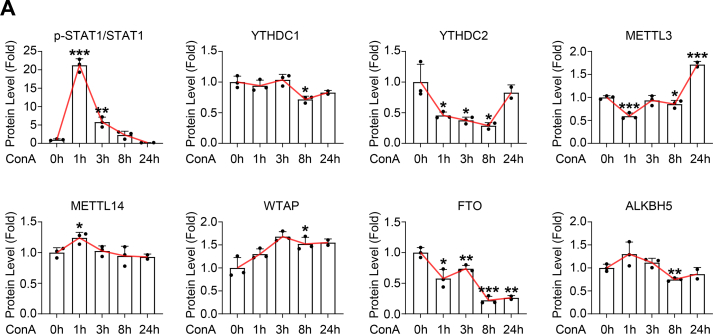
The changes of protein level in the m^6^A family during ConA-induced hepatitis. **(A)** Immunoblot analysis of liver tissues with m^6^A family and representative inflammatory pathway antibodies, followed by gray analysis using image J from Fig. 1A. Data are presented as mean ± SD; ∗*P <* 0.05, ∗∗*P <* 0.01, ∗∗∗*P <* 0.001 compared to the control group of WT ConA 0 h, based on two-side Student's *t*-test.

To investigate the correlation between m^6^A regulators and immune-mediated hepatitis, we initially examined the expression of YTHDF1 and other m^6^A regulators during ConA-induced liver injury. We observed a dramatic reduction in the protein expression of YTHDF1, YTHDF2 and YTHDF3 from 1 h to 8 h, followed by a sudden upregulation at 24 h (Fig. 1A, B). This expression pattern of YTHDF1, YTHDF2 and YTHDF3 appeared to be opposite to the p-STAT3 and p-STAT1 counterparts, two earliest activated markers of the inflammatory signaling pathway in ConA-induced hepatitis,^25^ as well as p-IKBα and p-ERK counterparts, suggesting that YTHDF1, YTHDF2 and YTHDF3 may serve as early warning signals for inflammation (Fig. 1A, B). In contrast, other m^6^A regulators, including METTL3, METTL14, WTAP, FTO, ALKBH5, YTHDC1, and YTHDC2, exhibited different protein expression patterns during ConA-induced hepatitis (Fig. 1A; Fig. S1A).

To identify the cell type responsible for the turnover of YTHDF1, YTHDF2, and YTHDF3 proteins during hepatitis, the immunohistochemical staining was conducted. Results confirmed a significant decrease in protein levels in hepatocytes during the early stage of hepatitis, which gradually increased later (Fig. 1C). Although, the mRNA levels of YTHDF1, YTHDF2, and YTHDF3 did not align with protein changes, consistent with previous studies (GSE45413 and GSE17184), indicating that mRNA expression of these regulators either increased slightly or remained relatively stable during early hepatitis post-ConA challenge compared to the controlled mice (Fig. 1E, F). Our RNA-Seq results further revealed only slight increases or no changes in mRNA levels of m^6^A regulators, including YTHDF1, YTHDF2, and YTHDF3, after the ConA challenge for 8 h (Fig. 1D). In conclusion, the rapid turnover of YTHDF1, YTHDF2, and YTHDF3 proteins during early ConA-induced hepatitis emerges as a hallmark of risk, suggesting their potential as crucial inflammation indicators in immune-mediated hepatitis.

### Deficiency of YTHDF1, not YTHDF3, exacerbates ConA-induced liver injury and mortality in mice

**Figure 2:**
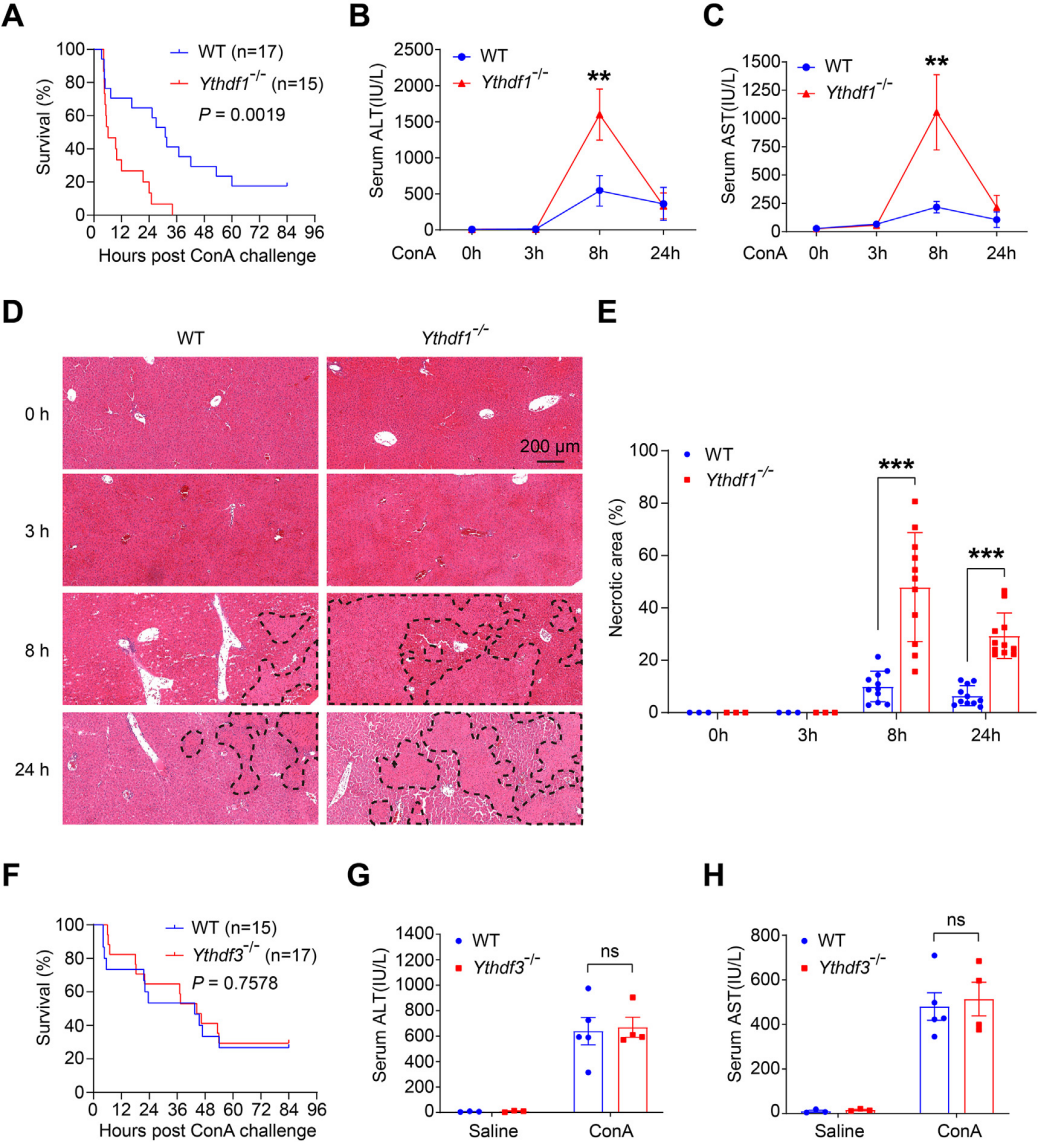
Deletion of YTHDF1, not YTHDF3, exacerbates ConA-induced liver injury and mortality in mice. **(A)** The mortality rate of YTHDF1 knockout (*Ythdf1*^−/−^) and wildtype littermates (WT) male mice at various time points after receiving an intravenous lethal dose of ConA (20 mg/kg), *n* = 15–17 per group. **(B**–**E)***Ythdf1*^−/−^ and WT male mice were administrated with ConA (8 mg/kg) or saline by intravenous injection, *n* = 3–5 per group. Serum samples or liver tissues were collected at 0 h, 3 h, 8 h, and 24 h after ConA injection. Measurement of Serum ALT (B) and AST (C) levels. H&E staining analysis of liver sections, Scale bar = 200 μm (D), the necrotic area was analyzed by image J (E). **(F)** The mortality rate of *Ythdf3*^−/−^ and WT male mice at various time points after receiving an intravenous lethal dose of ConA (20 mg/kg), *n* = 15–17 per group. **(G, H)***Ythdf3*^−/−^ and WT male mice were intravenously injected with ConA (8 mg/kg) or saline for 8 h, and serum samples were collected for the measurement of ALT (G) and AST (H) levels, *n* = 3–5 per group. Data are presented as mean ± SD; ∗*p <* 0.05, ∗∗*p <* 0.01, ∗∗∗*p <* 0.001, ns, not significant, compared with two groups of the corresponding time-point, based on two-sided Student's *t*-test. The survival curves are analyzed by the Log-rank test.

**Fig. S2:**
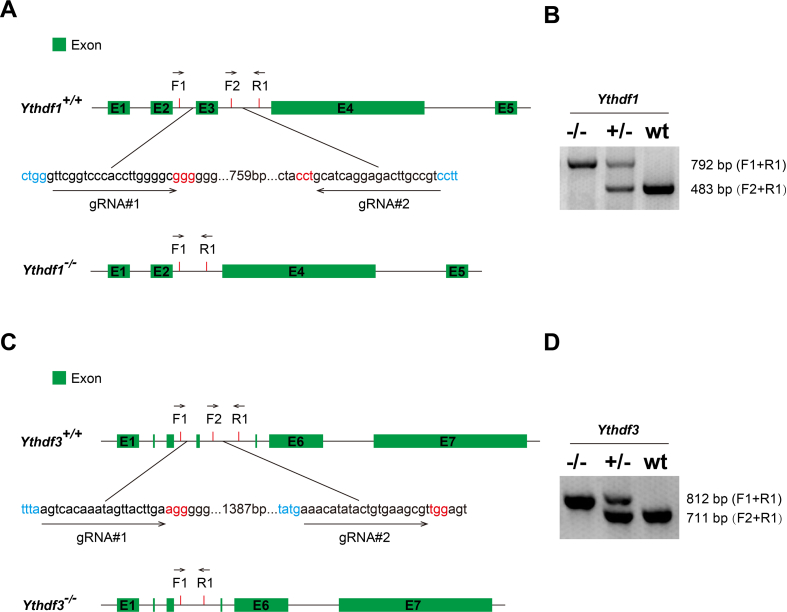
The schematic diagram for generating YTHDF1 and YTHDF3 deficiency mice. **(A)** Schematic image of targeting strategy for generating YTHDF1 deficiency mice based on CRISPR/Cas9 technology. **(B)** Genotype identification of tail in *Ythdf1*^*−/−*^, *Ythdf1*^*+/−*^, and wide type (wt, *Ythdf1*^*+/+*^) mouse. **(C)** Schematic image of targeting strategy for generating *Ythdf3* deficiency mice based on CRISPR/Cas9 technology. **(D)** Genotype identification of *Ythdf3*^*−/−*^, *Ythdf3*^*+/−*^, and wide type (wt, *Ythdf3*^*+/+*^) mouse. F1, forward primer 1. F2, forward primer 2. R1, reverse primer 1. gRNA, single guide RNA.

To explore the role of YTHDF1, YTHDF2, and YTHDF3 in experimental T cell-mediated hepatitis, the YTHDF1 or YTHDF3 deficient mice were generated using CRISPR/Cas9 technology (Fig. S2A–D). It is worth noting that YTHDF2 deficiency resulted in female infertility, preweaning lethality, and male hypo-fertile in mice.^26^ First, we intravenously injected ConA (20 mg/kg) into male WT, *Ythdf1*^−/−^, or *Ythdf3*^−/−^ mice, and monitored the survival rate of the mice every 2 h up to 84 h post the ConA administration. Notably, the survival rate of ConA-treated *Ythdf1*^−/−^ mice was dramatically lower than that of ConA-treated WT littermates throughout the observation period (Fig. 2A). By 36 h after ConA injection, all *Ythdf1*^−/−^ mice had succumbed, while approximately 40% of WT mice remained alive, with 20% surviving until the end of observation. However, no significant difference in survival rate was observed between *Ythdf3*^−/−^ mice and WT mice (Fig. 2F).

Subsequently, a sublethal ConA dose (8 mg/kg) was administered to the knockout mice and WT littermates, followed by biochemical and histological evaluation of liver injury. As expected, the serum levels of ALT and AST, established markers of liver injury, peaked at 8 h post-ConA injection. Consistent with the mortality findings, *Ythdf1*^−/−^ mice exhibited higher serum ALT and AST levels during the 8 h post-ConA treatment compared to WT mice (Fig. 2B, C), while *Ythdf3*^−/−^ mice showed no significant difference (Fig. 2G, H). Additionally, histological analysis revealed more pronounced hepatic necrosis in *Ythdf1*^−/−^ mice than in WT mice at 8 h and 24 h post-ConA administration (Fig. 2D, E). These results indicate that YTHDF1 deficiency, rather than YTHDF3, increase the susceptibility to ConA-induced hepatotoxicity and mortality.

### YTHDF1 deficiency exacerbates hepatic inflammatory response via ERK and NF-κB pathway

**Figure 3:**
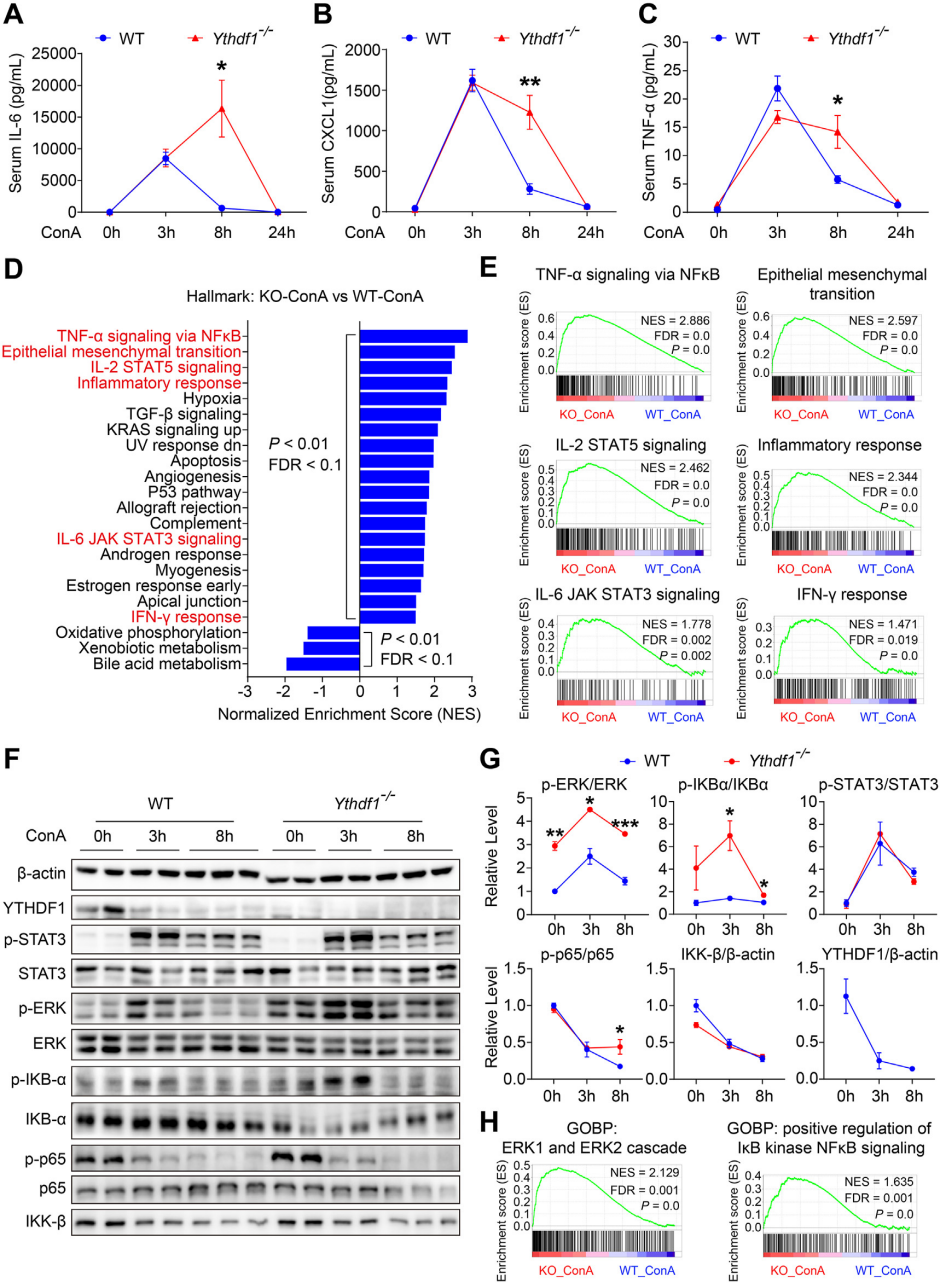
YTHDF1 deficiency aggravates hepatic inflammatory response via ERK and NF-κB pathway. **(A**–**C)** Serum samples collected at 0 h, 3 h, 8 h, and 24 h after ConA (8 mg/kg) injection from Fig. 2B and C was measured for IL-6 (A), CXCL1 (B), and TNF-α (C) levels, *n* = 3–5 per group. **(D)** Liver tissues of *Ythdf1*^−/−^ (KO) and WT mice collected at 8 h after ConA (8 mg/kg) treatment were extracted for RNA, followed by RNA-Seq analysis, *n* = 3–4 per group. GSEA analysis for RNA-Seq data identified the significant up-regulation or down-regulation gene sets of hallmarks mediated by YTHDF1 deletion upon ConA challenge for 8 h. **(E)** Representative hallmark pathways were shown for GSEA analysis. **(F, G)** Liver tissues harvested at 0 h, 3 h, and 8 h after ConA (8 mg/kg) treatment were immunoblot analyzed for the inflammatory pathway (F), followed by gray analysis using image J (G). **(H)** GSEA analysis of GOBP gene sets showed over-activation of ERK and NF-κB pathway mediated by YTHDF1 deletion upon ConA (8 mg/kg) challenge for 8 h. Data are presented as mean ± SD; ∗*p <* 0.05, ∗∗*p <* 0.01, ∗∗∗*p <* 0.001 compared with two groups of the corresponding time-point, based on unpaired two-sided Student's *t*-test. NES, normalized enrichment score. FRD, false discovery rate. KO, *Ythdf1*^*−/−*^.

Inflammatory cells, particularly the T lymphocytes, are crucial to the pathogenesis of both clinical AIH and ConA-induced experimental hepatitis.^4^ ConA, a potent T cell mitogen, activates T cells and induces inflammatory storms in mice, resulting in elevated serum cytokine levels, including IFN-γ, IL-6, TNF-α, and CXCL1.^5^^,^^6^ To elucidate the mechanisms underlying aggravated liver injury and mortality in YTHDF1-deficient mice following ConA injection, we analyzed serum cytokines (IL-6, CXCL1, and TNF-α) at various time points. As data showed, inflammatory mediators were rapidly increased at 3 h post-ConA both in *Ythdf1*^−/−^ mice and WT mice, which occurred earlier than hepatotoxicity, and *Ythdf1*^−/−^ mice exhibited consistently high levels of IL-6, CXCL1, and TNF-α until 8 h post-ConA compared to WT mice (Fig. 3A–C). Moreover, we performed transcriptome-wide RNA sequencing (RNA-seq) on liver tissue collected from *Ythdf1*^−/−^ mice and WT mice treated with ConA (8 mg/kg) or saline for 8 h. GSEA analysis in hallmark gene sets revealed that the inflammation-related signaling pathways were markedly over-activated in *Ythdf1*^−/−^ mice in contrast to WT mice during the ConA challenge, represented by the pathways of TNF-α signaling via NF-κB, epithelial–mesenchymal transition, IL-2 STAT5 signaling, inflammatory response, IL-6 JAK STAT3 signaling, and IFN-γ response (Fig. 3D, E). These findings suggest that YTHDF1 deficiency exacerbates hepatic inflammatory response during ConA-induced hepatitis. IL-6-STAT3 signaling, TNF-α–NF–κB signaling, and ERK signaling are essential for ConA-induced hepatitis through T-cell activation and hepatocyte destruction.^27^, ^28^, ^29^ Therefore, we investigated the activation of the STAT3, NF-κB, and ERK signal pathways. Our results revealed no differences in the phosphorylation status of STAT3 and p65 between WT and *Ythdf1*^−/−^ mice, whereas the phosphorylation levels of ERK and IκB-α were higher in *Ythdf1*^−/−^ mice than in WT mice after ConA administration (Fig. 3F, G). Consistent with these findings, GSEA analysis of GOBP (Gene Ontology Biological Process) gene sets indicated increased activation in pathways of ERK1/ERK2 cascade and positive regulation of IKK NF-κB signaling in ConA-treated *Ythdf1*^−/−^ mice compared to WT mice (Fig. 3H), supporting the over-activation of ERK and IκB-α signaling in ConA-treated *Ythdf1*^−/−^ mice. Collectively, these data demonstrate that YTHDF1 deficiency exacerbates the cytokine storm and hepatic inflammatory response, mainly through ERK and NF-κB pathways in ConA-induced hepatitis.

### Hepatic-specific overexpression or knockout of YTHDF1 is redundant in ConA-induced hepatitis

**Figure 4:**
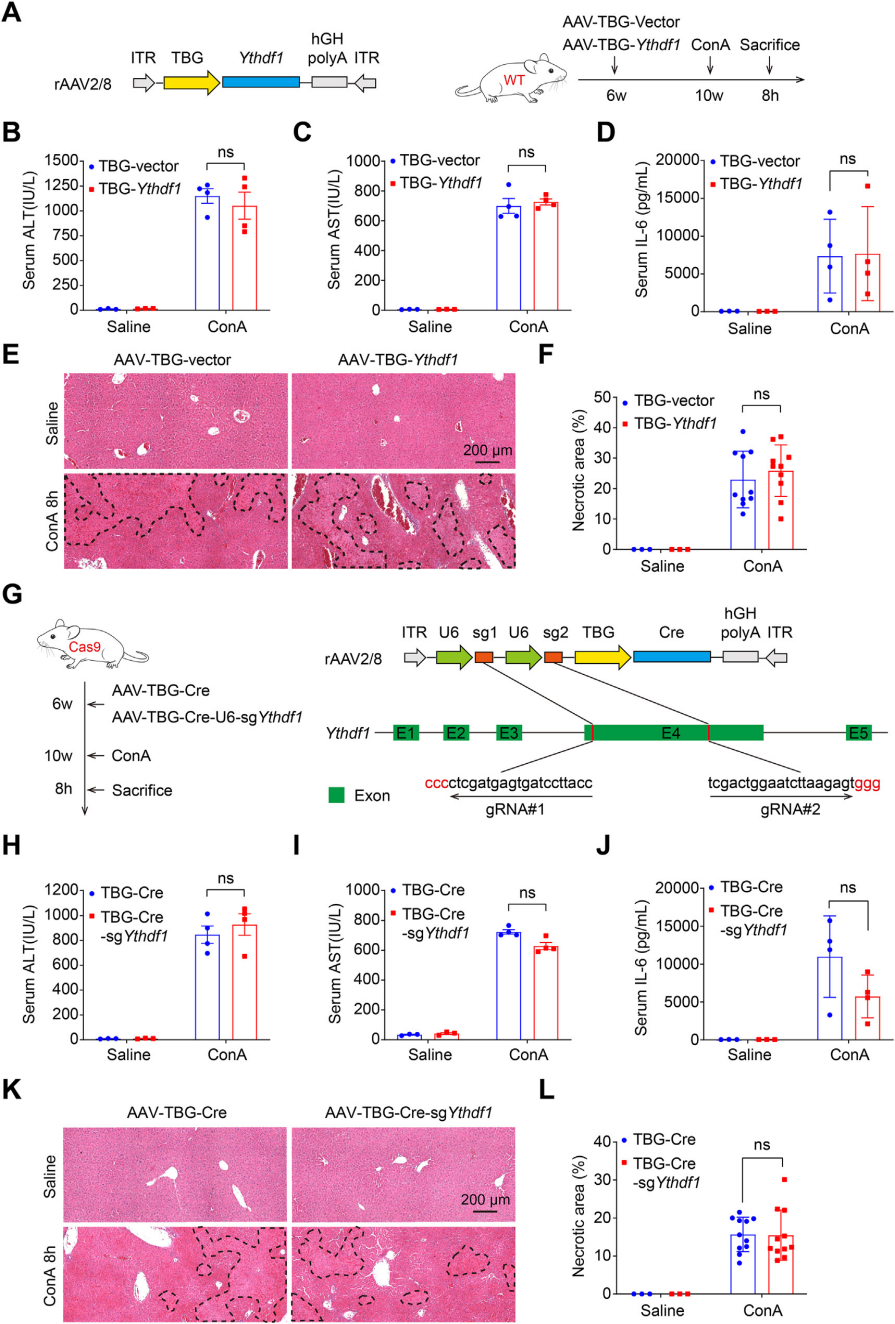
Hepatic-specific overexpression or deletion of YTHDF1 is redundant in ConA-induced hepatitis. **(A**–**F)** Schematic image of a mouse model for generating hepatic specific overexpression via TBG-driven mouse *Ythdf1* rAAV2/8 virus. Six weeks old C57BL/6 WT male mice were intravenously injected with rAAV2/8 virus. Four weeks after the virus injection, these mice were challenged by ConA (10 mg/kg) or saline for 8 h *n* = 3–5 per group (A). Serum samples were measured for ALT (B), AST (C), and IL-6 (D) levels. Liver samples were analyzed for H&E staining, Scale bar = 200 μm (E), followed by necrotic area analysis (F). **(G**–**L)** Schematic image of a mouse model for generating hepatic specific knockout of *Ythdf1* via AAV-CRISPR/Cas9 technology. Six weeks old transgenic Cre-dependent Cas9 male mice, tagged with upstream flox-STOP-flox element, were intravenously injected with rAAV2/8 virus, in which Cre was driven by liver-specific TBG promoter and two sgRNAs were driven by U6 promoters. Four weeks after virus injection, these mice were challenged by ConA (10 mg/kg) or saline for 8 h *n* = 3–4 per group (G). Serum samples were measured for ALT (H), AST (I), and IL-6 (J) levels. Liver samples were analyzed for H&E staining, Scale bar = 200 μm (K), followed by necrotic area analysis (L). Data are presented as mean ± SD; ∗*p <* 0.05, ∗∗*p <* 0.01, ∗∗∗*p* *<* 0.001, ns, not significant. ITR, inverted repeat sequence. TBG, thyroid binding globulin promoter.

Because of the observations in the dramatic decrease and subsequent increase in hepatic YTHDF1 protein during the early and late stages of ConA-induced hepatitis, we investigated the involvement of YTHDF1 in hepatocytes in the ConA-mediated inflammatory storm and hepatotoxicity. To this end, we generated hepatocyte-specific YTHDF1-overexpressing mice and YTHDF1-knockout mice by using AAV technology (Fig. 4A and G).

**Fig. S3:**
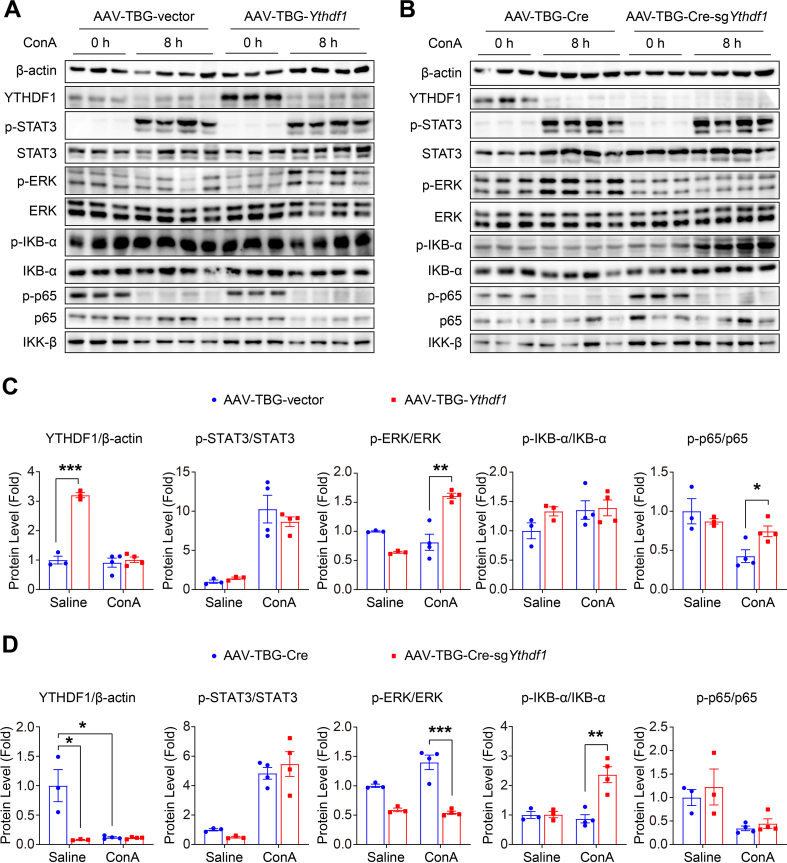
The hepatic alterations of signaling pathways during ConA challenge. Liver samples from Fig. 4 were analyzed by immunoblotting of inflammatory pathway **(A, B)**, followed by gray analysis **(C, D)**. Data are presented as mean ± SD; ∗*P <* 0.05, ∗∗*P <* 0.01, ∗∗∗*P* *<* 0.001.

Surprisingly, after the ConA injection, there were no differences in serum ALT, AST, IL-6 levels, or hepatic necrosis areas among control mice and those with hepatic-specific YTHDF1 overexpression or knockout (Fig. 4B–F, H–L). This suggests that hepatic YTHDF1 does not impact hepatocyte death or T cell activation-mediated inflammatory storms in ConA-induced hepatitis. However, Western blotting analysis of the inflammatory signaling pathway revealed that hepatic YTHDF1 overexpression enhanced ERK phosphorylation compared to control mice after ConA treatment, while other signals showed no significant changes (Fig. S3A, C). Abnormal signals were also observed in ConA-treated hepatic-specific YTHDF1 knockout mice, with decreased phosphorylated levels of ERK (p-ERK), increased phosphorylated levels of IκB-α (p-IκB-α), and unchanged phosphorylated levels of STAT3 (p-STAT3) compared to ConA-treated control mice (Fig. S3B, D). These results indicate the redundant role of hepatic YTHDF1 in affecting hepatocyte death and serum inflammatory storms induced by ConA administration, indirectly suggesting the essential role of YTHDF1 in inflammatory cells for ConA-induced hepatitis.

### Hepatic infiltrative inflammatory cells are over-activated in YTHDF1 deficiency mice during the ConA challenge

**Figure 5:**
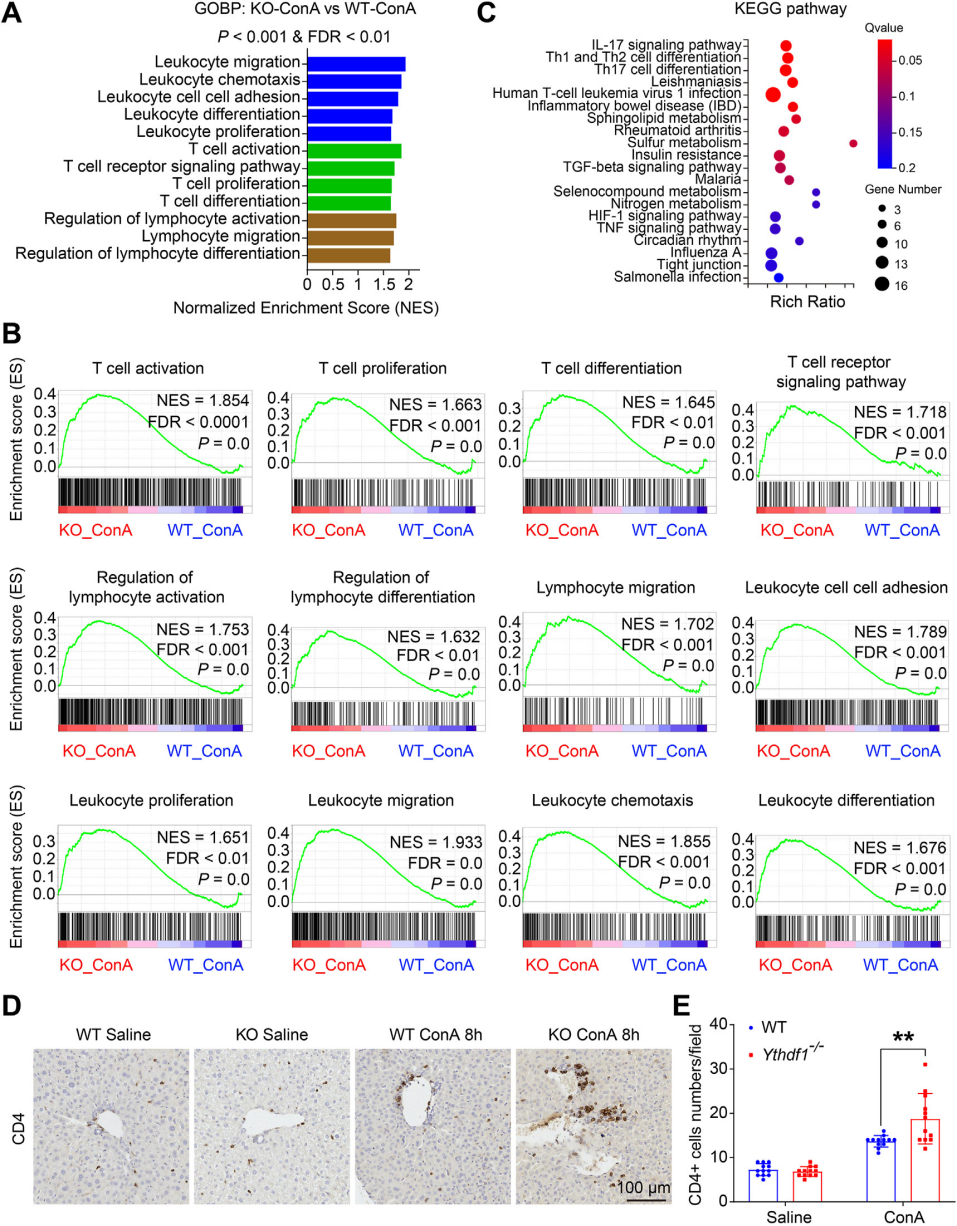
Hepatic infiltrative inflammatory cells are over-activated in YTHDF1-deficient mice during the ConA challenge. **(A, B)** RNA extracted from liver tissues of *Ythdf1*^−/−^ (KO) and WT male mice post ConA (8 mg/kg) treatment for 8 h were conducted for RNA-Seq analysis. GSEA analysis of RNA-Seq data by enriching GOBP gene sets indicated that hepatic infiltrative leukocyte, lymphocyte, and T cell were over-activated in *Ythdf1*^−/−^ mice after ConA challenge compared with WT counterpart (A), and the enrichment plots were showed (B). **(C)** KEGG enrichment for hepatic differential genes between *Ythdf1*^−/−^ and WT mice post ConA treatment. **(D, E)** Immunostaining of liver sections with CD4 antibodies (D), followed by statistical analysis of CD4^+^ cells per image, *n* = 12 per group (E). Scale bar = 100 μm. Data are presented as mean ± SD; ∗∗*p <* 0.01. GOBP, gene ontology biological process. NES, normalized enrichment score. FRD, false discovery rate. KEGG, Kyoto Encyclopedia of genes and genomes. KO, *Ythdf1*^*−/−*^.

Hepatic infiltrative leukocytes play an essential role in ConA-induced hepatitis.^6^ After intravenous injection, ConA primarily accumulates in the liver, binding to sinusoidal endothelial cells and activating T cells, in which, along with cytokine and chemokine production, recruits inflammatory cells.^4^^,^^5^ This exacerbates hepatic inflammatory response and damage. Analyzing RNA-seq data using GSEA enrichment of GOBP pathways revealed over-activation of pathways related to migration, chemotaxis, cell adhesion, differentiation, and proliferation of leukocytes, particularly T cells, in ConA-treated *Ythdf1*^−/−^ mice compared to WT mice (Fig. 5A. B). KEGG pathway analysis of differentially expressed genes between *Ythdf1*^−/−^ mice and WT mice indicated enrichment in IL-17 signaling, Th1/Th2 cell differentiation, and Th17 cell differentiation (Fig. 5C), suggesting that YTHDF1 deficiency affects the T cell function in ConA-induced hepatitis. Immunohistochemical staining with anti-CD4 antibody confirmed significantly higher CD4^+^ cell numbers in the ConA-treated *Ythdf1*^*−/−*^ mice livers than in WT mice (Fig. 5D, E). Taken together, these results imply that YTHDF1 deficiency could increase the infiltration and activation of inflammatory cells, especially CD4^+^ T cells, after intravenous injection of ConA.

### YTHDF1 in hematopoietic cells is necessary for the suppression of the inflammatory response

**Figure 6:**
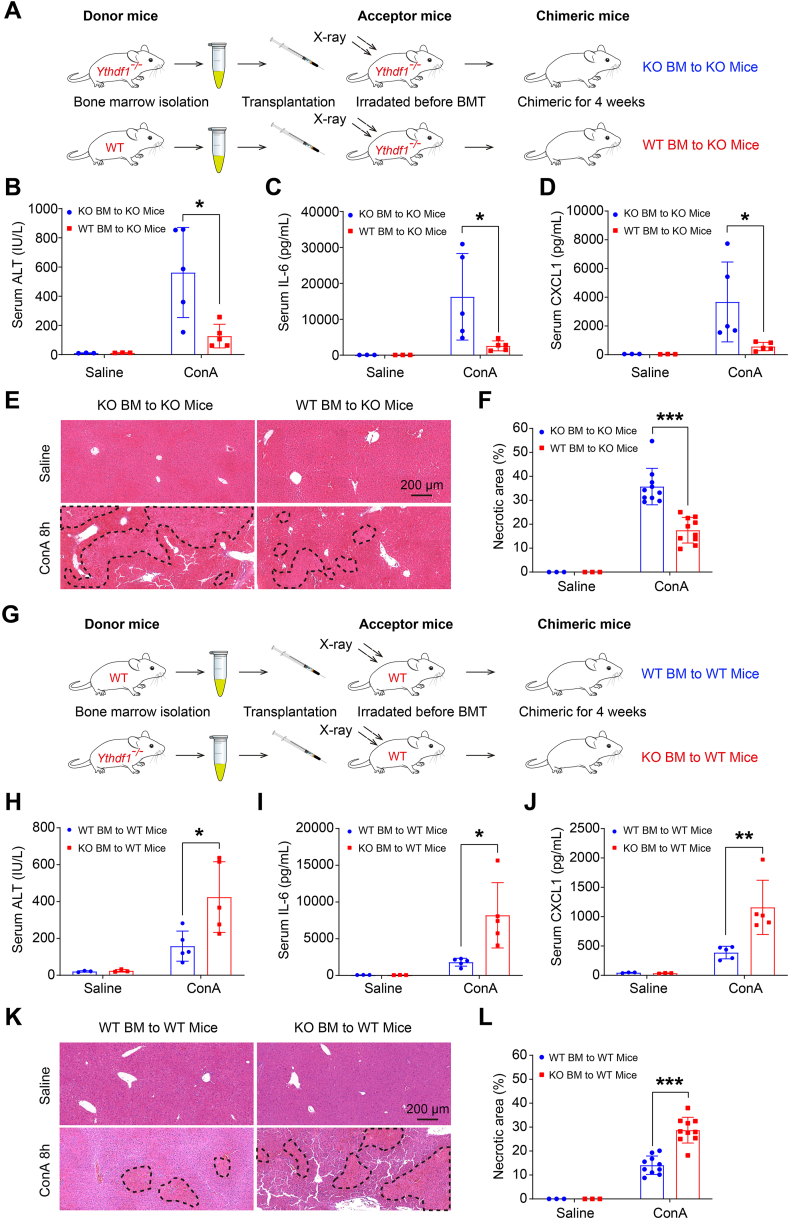
YTHDF1 in hematopoietic cells is necessary for the suppression of inflammatory response. **(A**–**F)** Eight weeks old male *Ythdf1*^−/−^ mice (KO) were irradiated with 8 Gy X-rays, and 6 h later, bone marrow cells (BM) from male donor *Ythdf1*^−/−^ or WT mice were transplanted into the irradiated mice with an intravenous injection at a number of 200E4 cells per mouse (A). Four weeks after transplantation, mice were intravenously injected with ConA (8 mg/kg) or saline for 8 h, *n* = 3–5 per group. Serum samples were measured for ALT (B), IL-6 (C), and CXCL1 (D) levels. Liver sections were analyzed by H&E staining (E) and the necrotic area was counted for statistical analysis (F). **(G**–**L)** Eight weeks old male WT mice were irradiated with 8 Gy X-rays, and 6 h later, bone marrow cells from male donor *Ythdf1*^−/−^ or WT mice were transplanted into the irradiated mice with an intravenous injection at a number of 200E4 cells per mouse (G). Four weeks after transplantation, mice were intravenously injected with ConA (8 mg/kg) or saline for 8 h, *n* = 3–5 per group. Serum samples were measured for ALT (H), IL-6 (I), and CXCL1 (J) levels. Liver sections were analyzed by H&E staining (K) and the necrotic area was counted for statistical analysis (L). Data are presented as mean ± SD; ∗*p <* 0.05, ∗∗*p <* 0.01, ∗∗∗*p <* 0.001. BMT, bone marrow transplantation. KO, *Ythdf1*^*−/−*^.

**Fig. S4:**
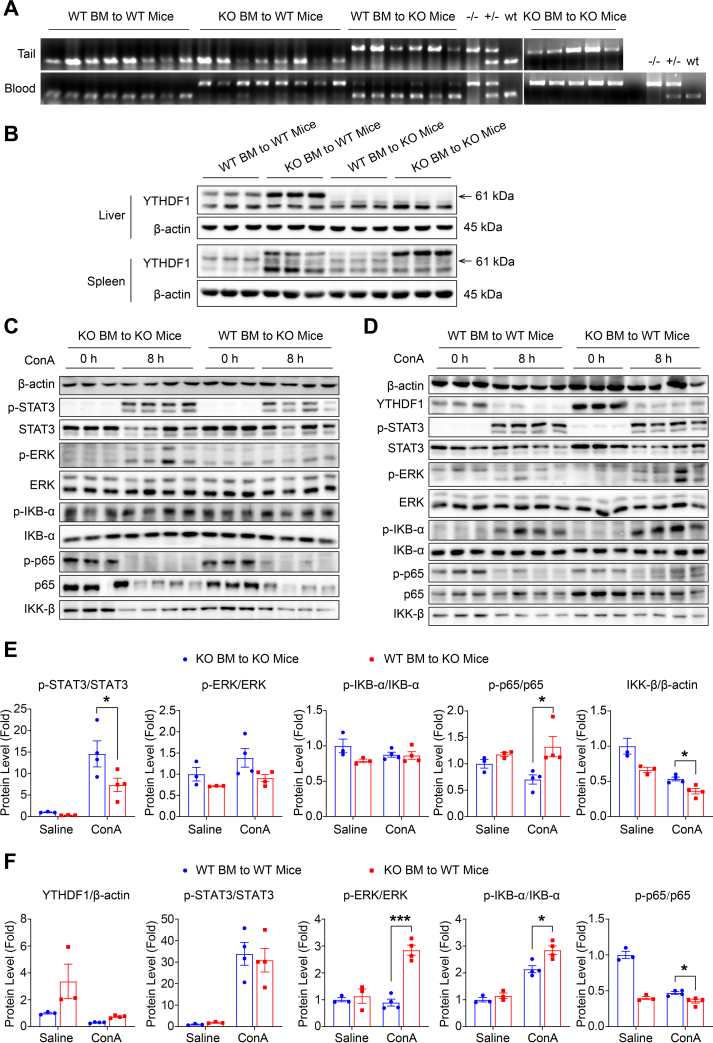
Identification of bone marrow transplantation in mice and the hepatic alterations of signaling pathways during ConA challenge in BMT mice. **(A)** Genotype identification of tail and blood samples in chimeric mice. **(B)** Western blotting of liver and spleen samples with YTHDF1 antibody in chimeric mice. The protein of liver samples from Fig. 6 was immunoblotted with inflammatory pathway antibodies **(C, D)**, followed by gray analysis **(E, F)**. Data are presented as mean ± SD; ∗*P <* 0.05, ∗∗*P <* 0.01, ∗∗∗*P <* 0.001. BM, bone marrow cells. KO, *Ythdf1*^*−/−*^. −/−, *Ythdf1*^*−/−*^. +/−, *Ythdf1*^*+/−*^.

While YTHDF1 in hepatocytes was redundant during T cell-mediated hepatitis, and *Ythdf1*^−/−^ mice exhibited heightened hepatic infiltration and immune cell activation, accompanied by an exacerbated inflammatory response, we investigated YTHDF1's role in hematopoietic cells using chimeric mice. We performed adoptive transfer of WT or *Ythdf1*^−/−^ bone marrow into lethally irradiated *Ythdf1*^−/−^ recipient mice (Fig. 6A). Four weeks post-transplantation, we confirmed transplantation efficiency (Fig. S4A, B), and ConA was administered to both chimeric strains for 8 h, followed by serum and liver sample collection. Remarkably, *Ythdf1*^−/−^ mice with WT bone marrow showed reduced liver injury, lower serum ALT levels, and decreased hepatic necrosis compared to those transplanted with *Ythdf1*^−/−^ bone marrow (Fig. 6B–E, F). Importantly, the replacement of WT bone marrow in *Ythdf1*^−/−^ mice significantly rescued serum inflammatory mediator levels (IL-6 and CXCL1) (Fig. 6C, D), indicating that YTHDF1 in hematopoietic cells is crucial for suppressing ConA-induced inflammatory cytokine and chemokine release. In parallel experiments, we observed suppressed phosphorylation levels of STAT3 and ERK in *Ythdf1*^−/−^ mice with bone marrow transplanted into WT counterparts (Fig. S4C, E). Conversely, adoptive transfer of WT or *Ythdf1*^−/−^ bone marrow into lethally irradiated WT recipient mice (Fig. 6G) revealed that WT mice receiving *Ythdf1*^−/−^ bone marrow were more susceptible to ConA-induced liver injury and inflammatory response, as evidenced by elevated serum levels of ALT, IL-6, and CXCL1, enhanced hepatic necrosis, and high levels of hepatic p-ERK and p-IκBα (Fig. 6H–L; Fig. S4D, F). These findings confirm that YTHDF1 in hematopoietic cells acts as a suppressor for the inflammatory response during ConA-induced hepatitis.

### YTHDF1 deletion in macrophages aggravates LPS-induced inflammation

**Figure 7:**
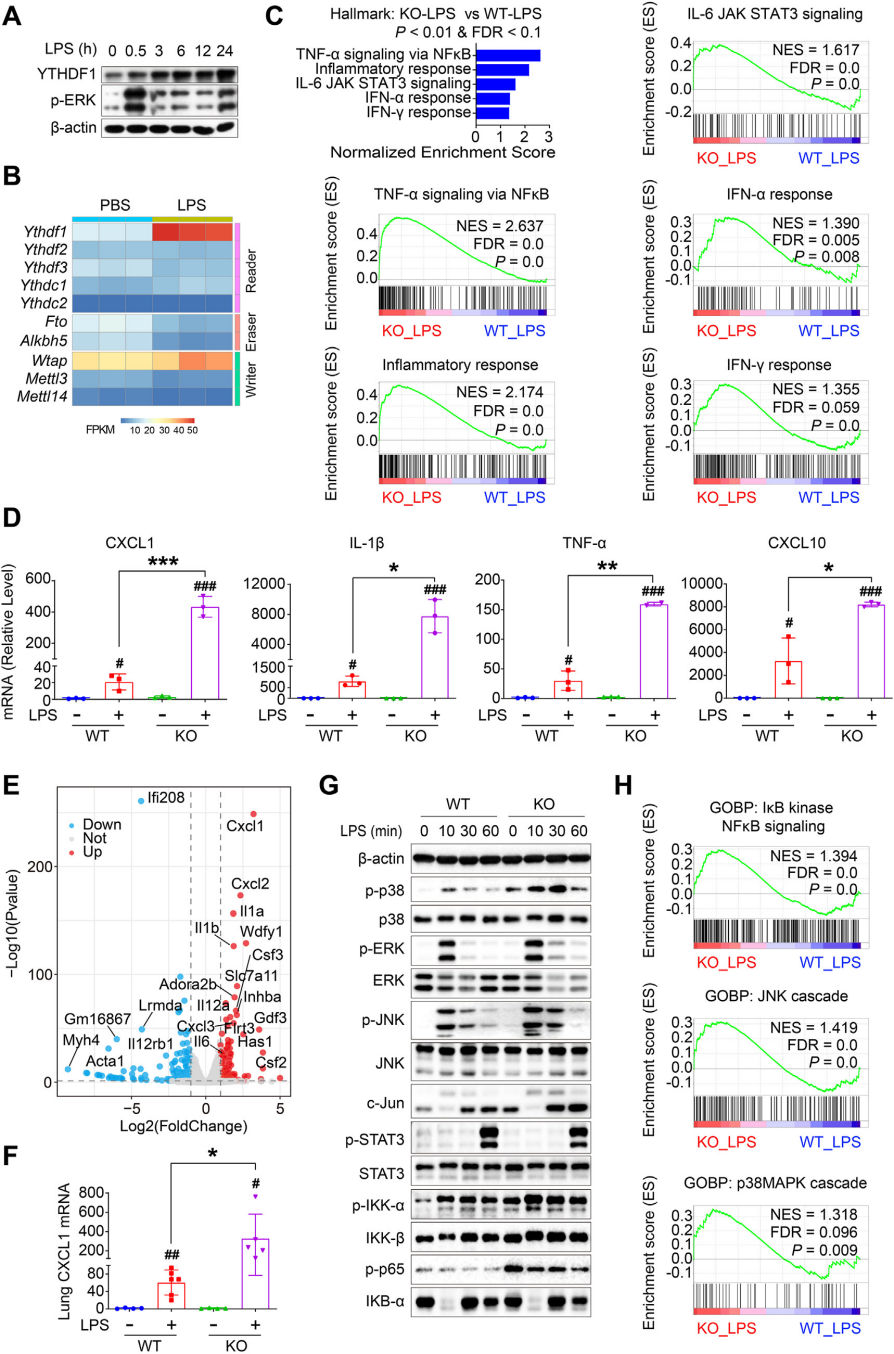
YTHDF1 deficiency in macrophages enhances LPS-induced inflammation. **(A)** Primary peritoneal macrophages (PM) extracted from WT male mice were stimulated with LPS (1 μg/mL) for indicated times, followed by immunoblotting with YTHDF1 and p-ERK antibodies. **(B)** PM from WT mice was stimulated with LPS (1 μg/mL) or vehicle for 6 h, followed by RNA-Seq analysis and heatmap analysis of the m^6^A family. *n* = 3 per group. **(C)***Ythdf1*^−/−^ (KO) or WT PM were stimulated with LPS (1 μg/mL) for 6 h, followed by RNA-Seq analysis and GSEA enrichment in hallmark gene sets, showing the differentially expressed gene sets. **(D)** RT-qPCR analysis of inflammatory factors including CXCL1, IL-1β, TNF-α, and CXCL10 in *Ythdf1*^−/−^ (KO) or WT PM challenged by LPS (1 μg/mL) or vehicle for 6 h *n* = 3 per group. **(E)** Volcano plot analysis showed the differentially expressed genes between *Ythdf1*^−/−^ (KO) and WT PM (KO versus WT) after LPS (1 μg/mL) stimulation for 6 h. **(F)** Eight-week-old male WT or *Ythdf1*^−/−^ (KO) mice were peritoneally injected with LPS (10 mg/kg) or vehicle for 6 h, followed by the collection of lung samples for RT-qPCR analysis of CXCL1. *n* = 4–6 per group. **(G)** The protein of PM post LPS challenge (1 μg/mL) for indicated times was immunoblotted with inflammatory pathway antibodies. **(H)** GSEA enrichment in GOBP gene sets showed that the NF-κB, JNK, and p38 MAPKs pathways were over-activated in LPS-treated *Ythdf1*^−/−^ (KO) PM compared with WT PM. Data are presented as mean ± SD; ∗*p <* 0.05, ∗∗*p <* 0.01, ∗∗∗*p <* 0.001, compared with two indicated groups; ^#^*p <* 0.05, ^##^*p <* 0.01, ^###^*p <* 0.001, compared with WT vehicle group. PM, peritoneal macrophages. NES, normalized enrichment score. FRD, false discovery rate. MAPKs, mitogen-activated protein kinases. GOBP, gene ontology biological process. KO, *Ythdf1*^*−/−*^.

Indeed, unlike the liver in ConA-induced hepatitis, the LPS challenge increased both YTHDF1 protein and mRNA levels in PM (Fig. 7A, B), indicating YTHDF1's potential role as an inflammatory regulator. Therefore, we hypothesized that YTHDF1 deletion in macrophages would enhance inflammatory responses, mirroring aggravated ConA-induced hepatitis in *Ythdf1*^−/−^ mice and bone marrow chimeric mice. As expected, knockout of YTHDF1 in macrophages enhanced the inflammatory response induced by LPS, as demonstrated by over-activated inflammation-related pathways (including TNF-α signaling via NF-κB pathway, inflammatory response pathway, IL-6/JAK/STAT3 signaling pathway, IFN-α response pathway, and IFN-γ response pathway) in LPS-treated *Ythdf1*^−/−^ macrophages compared with WT counterparts, which were assessed through GSEA enrichment of hallmark gene sets (Fig. 7C). RT-qPCR analysis revealed significantly elevated mRNA levels of inflammatory mediators (CXCL1, IL-1β, TNF-α, and CXCL10) in YTHDF1-knockout cells under LPS challenge (Fig. 7D). The volcano plot analysis highlighted the increased expression of inflammatory mediators (CXCL1, CXCL2, IL-1α, IL-1β, and IL-6) in LPS-treated *Ythdf1*^−/−^ PM (Fig. 7E). Moreover, the *Ythdf1*^−/−^ mice were more susceptible to LPS-mediated sepsis *in vivo*, evidenced by increased CXCL1 mRNA expression in the lung (Fig. 7F).

To examine the underlying mechanism by which YTHDF1 deficiency promotes inflammation in macrophages, we then checked the activation of mitogen-activated protein kinases (MAPKs), NF-κB, and STAT3 signal pathways. The immunoblot analysis of signal pathways indicated that *Ythdf1*^−/−^ macrophages had higher phosphorylation status of p38, IKK-α, and p65 than WT counterparts both in basal level and early stage of LPS treatment (Fig. 7G), suggesting that YTHDF1 deficiency rendered myeloid cells more sensitive to inflammatory stimulation. Additionally, GSEA enrichment indicated that the NF-κB, JNK, and p38 MAPK pathways were over-activated in LPS-treated *Ythdf1*^−/−^ PM compared with WT PM (Fig. 7H). Collectively, YTHDF1 deficiency in macrophages exacerbated LPS-induced inflammation, highlighting YTHDF1's role in suppressing the inflammatory response in myeloid cells.

## Discussion

**Figure 8:**
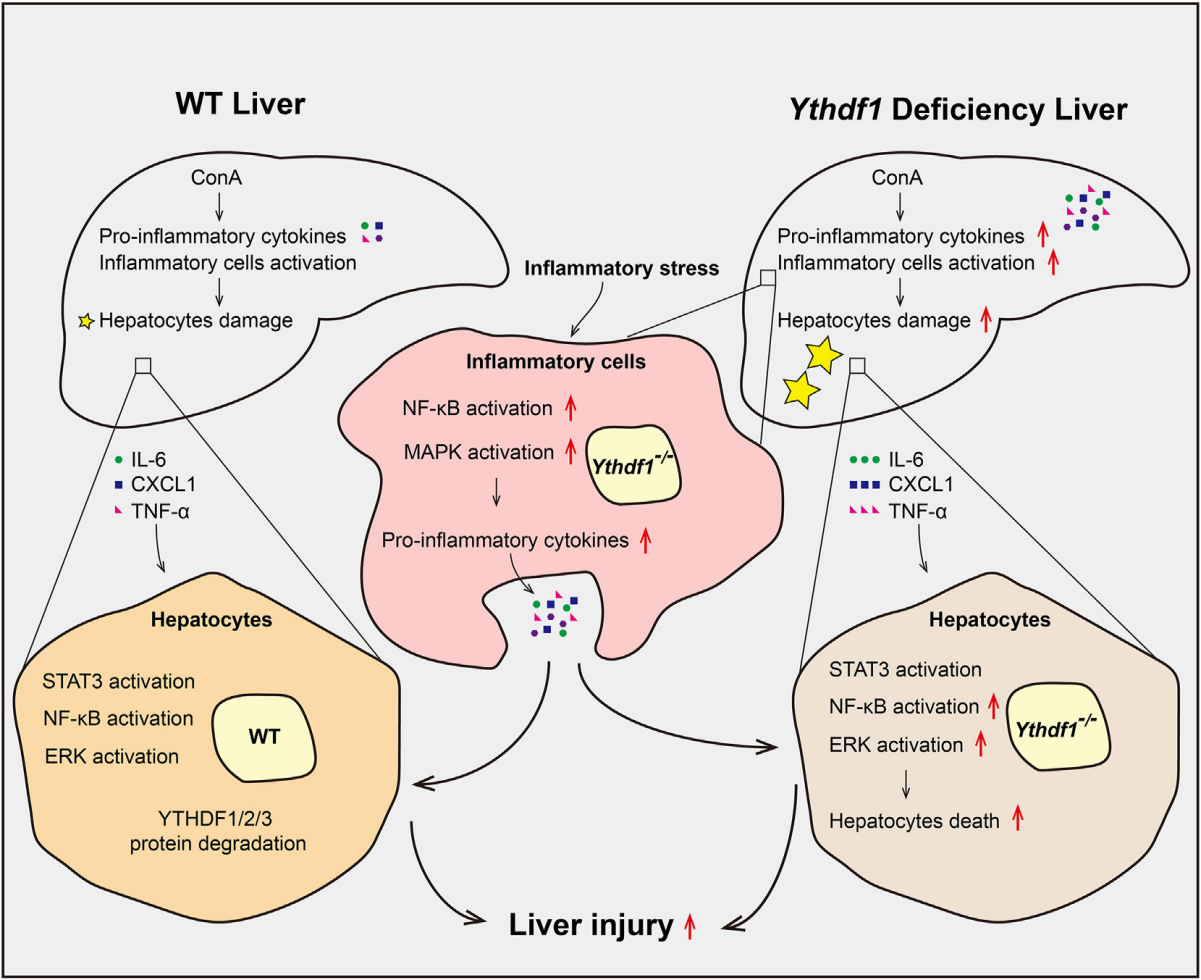
Schematic summary for the mechanism by which YTHDF1 shapes immune-mediated hepatitis via regulating inflammatory cell recruitment and response.

In recent studies, m^6^A modification and its regulators have emerged as potential contributors to the pathogenesis of inflammatory diseases and autoimmune disorders.^11^^,^^13^^,^^14^^,^^19^^,^^20^ Based on our findings, we proposed a model in which YTHDF1 shapes immune-mediated hepatitis via regulating inflammatory cell recruitment and response (Fig. 8). Initially, we investigated the protein expression of m^6^A readers, YTHDF1, YTHDF2, and YTHDF3, during the early stage of ConA-induced hepatitis and found that they were dramatically decreased in the liver. Notably, the deficiency of YTHDF1, but not YTHDF3, exacerbated hepatic injury, marked by heightened cytokine production and inflammatory cell infiltration during ConA-induced hepatitis. Surprisingly, hepatic-specific knockout or overexpression of YTHDF1 showed no significant impact on hepatic injury and cytokine release, suggesting a redundant role of YTHDF1 in hepatocytes during ConA-induced hepatitis. Instead, our findings highlighted the crucial role of YTHDF1 in hematopoietic cells, particularly in suppressing T cell-mediated liver injury and ConA-induced cytokine storms. Moreover, the absence of YTHDF1 in macrophages intensified LPS-induced inflammation, emphasizing its necessity in immune cells for regulating the inflammatory response. These results collectively suggest the immunomodulatory and anti-inflammatory role of YTHDF1 in T cell-mediated hepatitis.

Autoimmune hepatitis (AIH), characterized by an unclear etiology, is diagnosed using traditional biomarkers such as elevated serum aminotransferases (ALT and AST), IgG levels, and autoantibodies.^30^ Although novel noninvasive peripheral candidate biomarkers in autoimmune hepatitis have been recently described,^30^ histological evidence of liver biopsy is specific for identifying active disease and the state of histologic remission.^31^ Here, our study proposes m^6^A readers, including YTHDF1, YTHDF2, and YTHDF3, as potential diagnostic markers for AIH in humans. The rapid decrease and turnover of these proteins during ConA-induced hepatitis may serve as indicative markers of active disease and histologic remission in AIH. Additionally, the association of YTHDF1 overexpression with poor prognosis in hepatocellular carcinoma suggests its potential as a prognostic biomarker.^32^, ^33^, ^34^ However, further studies are required to understand their regulation in T cell-mediated hepatitis.

As an m^6^A reader, YTHDF1 plays a crucial role in inflammation, immunity, and tumorigenesis. YTHDF1 deficiency in mice has been linked to increase the antigen-specific CD8^+^ T cell antitumor responses,^35^ and the susceptibility for sepsis.^18^ Our study further supports the critical role of YTHDF1 in suppressing inflammation during T cell-mediated hepatitis. Despite a dramatic decrease in hepatic YTHDF1 protein during ConA-induced hepatitis, specific over-expression, or knockout in hepatocytes may not affect hepatic damage or cytokine release, implying redundancy. Instead, YTHDF1 deficiency sensitized inflammatory cells to ConA stimulation, leading to the exacerbated cytokine production, an aggravated hepatic inflammatory response, and increased hepatocyte cell death. These results indirectly suggest that YTHDF1 in hematopoietic cells may be necessary for T cell-mediated hepatitis.

Proinflammatory cytokines released from activated T cells and macrophages, including TNF-α, IFN-γ, IL-6, and CXCL1, are critical in ConA-induced hepatitis.^5^^,^^36^^,^^37^ In our findings, *Ythdf1*^−/−^ mice exhibited higher serum levels of these cytokines upon ConA challenge, indicating that YTHDF1 deficiency sensitizes inflammatory cells to ConA, intensifying cytokine production and exacerbating hepatic inflammation. Nuclear factor-kappa B (NF-κB) pathway and MAPKs pathway, including extracellular signal-regulated kinase, c-Jun NH2-terminal kinase (JNK), and p38 MAPKs,^38^ which were activated by inflammatory cytokines, played essential roles in liver pathological and physiological processes,^29^^,^^39^ as well as the pathogenesis of AIH.^40^^,^^41^ The activation of NF-κB and MAPKs signaling after ConA treatment in *Ythdf1*^−/−^ mice further emphasizes the role of YTHDF1 in suppressing inflammatory responses.

In human autoimmune hepatitis, CD4^+^ T cells predominate in mononuclear infiltration.^2^ A recent report highlighted the close association of the YTH domain family with immune cell infiltration, encompassing CD4^+^ T cells, CD8^+^ T cells, B cells, dendritic cells, macrophages, and neutrophils in hepatocellular carcinoma.^42^ Furthermore, studies have affirmed that dynamic m^6^A methylation in T lymphocytes, macrophages, and dendritic cells influences the immune response and antitumor immunity in liver disease.^43^ Our findings in a mouse model of immune hepatitis suggest that YTHDF1 plays a crucial role in suppressing inflammatory cell invasion, particularly CD4^+^ lymphocytes. The overactivated pathways in *Ythdf1*^−/−^ mice indicated the YTHDF1's significant role in regulating migration, chemotaxis, cell adhesion, differentiation, and proliferation of inflammatory cells during the immune response.

A recent study demonstrated that myeloid-cell-specific knockout of METTL14 sensitized mice to bacterial infection, highlighting the hematopoietic origin of the hyper-inflammatory response.^18^ We speculated that the hypersensitivity observed in ConA-treated *Ythdf1*^−/−^ mice is due to defects in hematopoietic cells. Indeed, bone marrow transplantation analysis supported that YTHDF1 deficiency in hematopoietic cells is the origin of the hypersensitive response during ConA-induced hepatitis. However, further research using T cells conditional knockout of YTHDF1 is warranted for exploring its role in T cell-mediated hepatitis. Additionally, the heightened sensitivity of YTHDF1-deficient macrophages to LPS-induced inflammation aligns with previous findings on the YTH domain family's regulation of infectious responses in macrophages.^18^^,^^44^, ^45^, ^46^

## Conclusions

Our study suggests that YTHDF1 plays a critical role in immune-mediated hepatitis by regulating inflammatory cell recruitment and response. Deficiency of YTHDF1, particularly in hematopoietic cells, amplifies inflammatory mediator production, leading to heightened hepatic inflammatory cell infiltration and exacerbated hepatic inflammatory response, ultimately causing severe liver injury. Thus, YTHDF1 emerges as a potential therapeutic target for immune-mediated hepatitis.

## Ethics declaration

All animal experiments adhered to the guidelines outlined in National Institute of Health Guide for the Care and Use of Laboratory Animals and received approval from the Ethics Committee of Xiamen University, Fujian, China. Animal protocols were further approved by the Institutional Animal Care and Use Committee of Xiamen University and strictly followed the standard veterinary practice as defined by the Xiamen University Laboratory Animal Center.

## Author contributions

Conceptualization: Hao Li and Yongyou Zhang. Data curation: Hao Li, Kailun Yu, and Xiandan Zhang. Funding acquisition: Yongyou Zhang. Investigation: Hao Li and Kailun Yu. Methodology: Hao Li, Kailun Yu, Xiandan Zhang, Jiawen Li. Software: Hao Li, Xusheng Deng and Siyu Zeng. Supervision: Yongyou Zhang. Visualization: Xiaoning Dong and JunRu Zhao. Validation: Huilong Hu. Writing original draft: Hao Li. Writing-review and editing: Yongyou Zhang.

## Funding

This work was supported by grants from the 10.13039/501100001809National Natural Science Foundation of China (No. 81772539, 81972238), and the Fundamental Research Funds for the Central Universities of China-Xiamen University (No. 20720180048).

## Conflict of interests

Yongyou Zhang is an editorial board member for Genes & Diseases and was not involved in the editorial review or the decision to publish this article. All authors declare that there are no competing interests.
